# A feedback constraint optimization method for intensity-modulated radiation therapy of nasopharyngeal carcinoma

**DOI:** 10.3892/ol.2015.3523

**Published:** 2015-07-23

**Authors:** YONGWU LI, XIAONAN SUN, QI WANG, QINXUAN ZHOU, BENXING GU, GUOZHI SHI, DONGLIANG JIANG

**Affiliations:** Department of Radiation Oncology, Sir Run Run Shaw Hospital, College of Medicine, Zhejiang University, Hangzhou, Zhejiang 310000, P.R. China

**Keywords:** feedback constraint optimization, nasopharyngeal carcinoma, radiation therapy, dose

## Abstract

Intensity-modulated radiation therapy (IMRT) is able to achieve good target conformance with a limited dose to organs at risk (OARs); however, IMRT increases the irradiation volume and monitor units (MUs) required. The present study aimed to evaluate the use of an IMRT plan with fewer segments and MUs, while maintaining quality in the treatment of nasopharyngeal carcinoma. In the present study, two types of IMRT plan were therefore compared: The direct machine parameter optimization (DMPO)-RT method and the feedback constraint DMPO-RT (fc_DMPO-RT) method, which utilizes compensative feedback constraint in DMPO-RT and maintains optimization. Plans for 23 patients were developed with identical dose prescriptions. Each plan involved synchronous delivery to various targets, with identical OAR constraints, by means of 7 coplanar fields. The average dose, maximum dose, dose-volume histograms of targets and the OAR, MUs of the plan, the number of segments, delivery time and accuracy were subsequently compared. The fc_DMPO-RT exhibited superior dose distribution in terms of the average, maximum and minimum doses to the gross tumor volume compared with that of DMPO-RT (t=62.7, 20.5 and 22.0, respectively; P<0.05). The fc_DMPO-RT also resulted in a smaller maximum dose to the spinal cord (t=7.3; P<0.05), as well as fewer MUs, fewer segments and decreased treatment times than that of the DMPO-RT (t=6.2, 393.4 and 244.3, respectively; P<0.05). The fc_DMPO-RT maintained plan quality with fewer segments and MUs, and the treatment time was significantly reduced, thereby resulting in reduced radiation leakage and an enhanced curative effect. Therefore, introducing feedback constraint into DMPO may result in improved IMRT planning. In nasopharyngeal carcinoma specifically, feedback constraint resulted in the improved protection of OARs in proximity of targets (such as the brainstem and parotid) due to sharp dose distribution and reduced MUs.

## Introduction

Treatment of complex head and neck cancers with intensity-modulated radiation therapy (IMRT) may facilitate improved survival and local disease control (1–3). IMRT conforms to the target and administers a limited radiation dose to the organs at risk (OAR). However, IMRT also results in increased whole-body exposure to low-dose radiation, an extended treatment time (proportional to the total number of segments required) and increased monitor units (MUs); therefore, treatment efficiency is reduced. Larger MUs also increase the incidence of radiation leakage, resulting in a higher risk of development of secondary cancers. An IMRT plan with fewer segments and MUs may overcome these treatment complications; however, it is difficult to optimize the IMRT plan whilst maintaining high quality treatment with good target conformance, improving the therapeutic ratio and reducing the risk of secondary cancer.

Hall and Wuu (4) reported that IMRT with a larger irradiation volume and a lower dose increased radiation-induced secondary cancer compared with conventional radiotherapy from ~1 to ~1.75% for patients surviving 10 years. From the forward IMRT plan initially advocated, to the inverse IMRT plan proposed by Bortfeld (5), planning quality and IMRT methods have markedly improved. Inverse IMRT is able to significantly improve the coverage of the planned target with a more uniform dose, while also decreasing the number of segments and reducing the MUs. Therefore, inverse IMRT continues to be widely used. There are two optimization methods for inverse IMRT: Intensity modulation (IM) fluence and direct step-and-shoot (DSS). The IM fluence optimization method, also known as the two-step optimization method, involves fluence optimization, followed by generation of the segment by combining the multi-leaf collimator (MLC) and other physical constraints. The segment generated sacrifices the quality of fluence optimization, and consequently, plan quality is not improved. Therefore, studies have proposed improved fluence optimization methods (6–8), including intensity limitation and segment smoothing; however, these changes are insufficient. For example, intensity limitation does not prevent increases in the small MU fluctuation during processing, and segment smoothing deteriorates plan quality when there is a steep dose gradient distribution. Matuszak *et al* (8) proposed the addition of the penalty function to the segment smoothing method in intensive modulation, in order to obtain a larger MU reduction. The second optimization method, DSS, is also known as the one-step optimization method. With the DSS method, the MLC and other physical constraint parameters are directly included in the optimization process, and no subsequent leaf sequencing is required (9–12). Furthermore, there is no detriment to treatment quality, and the plan is able to be directly executed. Based on the methods used, DSS may be further divided into direct machine parameter optimization (DMPO), in the case of a gradient descent algorithm; or direct aperture optimization (DAO), in the case of a simulated annealing algorithm. Since DSS results in improved plan quality and fewer segments than IM, it has become the preferred optimization method for inverse IMRT (5,9,10,13). Bedford and Webb (6) reported that the segment shape constraint function was able to reduce the calculating parameters of optimization, and combined with the utilization of a smooth and regular shape to the limit segments, reduced jaggedness was achievable. They also proposed a feedback optimization method to generate subsequent segment parameters based on the first segments, so that a simple and efficient plan may be created. Carlsson (12) reported a method of generating fewer, less jagged segments with a large area. This method was based on the shortest path of fluence gradient downwards; during the optimization process, irregular or small areas of segments were avoided, and when it was necessary to alter the dose, the segments exhibited improved conformal change. Pinnacle's white paper on DMPO mentioned the gradient descent method, but not the feedback method (13). In the present study, the method of DMPO with feedback constraints was applied. The feedback constraints reflected the dose distribution expected for the planned target and the mutual association between the geometric and topological structure between the target and OAR.

Studies by Dobler *et al* (9,10) revealed that, in the treatment of head and neck cancer, DMPO exhibited greater advantages and a wider application than IM. Nasopharyngeal carcinoma (NPC) is one of the most common types of head and neck cancer in South China, and the treatment of NPC requires complex planning as the dose distribution of the target and OAR contain and compete with each other. To improve the clinical outcome of NPC, an improved IMRT approach is required. The present study designed 2 types of IMRT plan for the treatment of NPC: The DMPO radiotherapy (DMPO-RT) method and the feedback constraint DMPO radiotherapy (fc_DMPO-RT) method. The plan quality, segment number and MU output of these 2 types of IMRT plan were generated using various methods and compared, in order to establish which strategy was superior.

## Materials and methods

### 

#### Patients

Twenty-three patients with NPC were treated at the Sir Run Run Shaw Hospital (Hangzhou, China) during the latter half of 2012. The median age of the patients was 57 years (range, 36–70 years), and additional patient characteristics are outlined in Table I. The present study was approved by the ethics committee of the Sir Run Run Shaw Hospital (Hangzhou, China) and all participating patients provided informed consent.

#### Immobilization and computed tomography (CT) scanning

Utilizing head thermoplastic immobilization (Q Fix Systems, Avondale, PA, USA), patients in the supine position underwent CT analysis, with a slice thickness of 3 mm using a SOMATOM Definition AS CT scanner (Siemens AG, Erlangen, Germany). The scan range extended from the orbital cavity to the sternoclavicular joint below.

#### Target and OAR delineation

The primary tumor plus grossly enlarged lymph nodes were defined as the gross tumor volume (GTV), any microscopic extensions of the GTV together with the regional lymphatics were defined as clinical target volume 1 (CTV_1_), and potential sites of microscopic extension were defined as clinical target volume 2 (CTV_2_). Based on the hospital data regarding the set-up position error, the appropriative margins of 6.0, 3.0 and 3.0 mm extensions of GTV, CTV_1_, and CTV_2_ were defined as planning target volume (PTV)70, PTV60 and PTV50, with prescription doses 70, 60 and 50.4 Gy, respectively. The modified PTV (mPTV) parameter was proposed to contain only 1 dose level by truncating at the point of overlap with higher-dose PTVs. Therefore, mPTV50 indicated PTV50 truncated at where PTV60 and PTV70 overlapped with PTV50, and mPTV60 indicated PTV60 truncated at where PTV70 overlapped with PTV60. OARs were defined and delineated according to the International Commission on Radiation Units and Measurements (ICRU) report 83 (14).

#### Design of treatment plan

Pinnacle v7.6 (Philips Medical Systems, Madison, WI, USA) treatment planning software (TPS) was used for IMRT planning. The Pinnacle DMPO optimizer is a RayOptimizer with the Nonlinear Programming Systems Optimization Laboratory at Stanford core, based on the gradient function. In every iteration, the DMPO optimizer uses the gradient of the objective function with respect to the optimization parameters (leaf positions and weights) to find an update of the parameters that improves the objective function; the result includes the machine parameter, allowing it to be directly delivered without additional conversion. It is important with the DMPO method to define a control point as iterative initials. In the present study, when a stage of optimization was completed, this stage was defined as the initial control point. Subsequently, the feedback constraints, which compensated for a hot or cold dose region on the planned target, were added. The feedback constraints also included areas that presented the mutual association between the geometric and topological structure of the target and organ that may compete with the dose. Optimization was recycled until the optimization outcome met the plan requirements.

**Figure 1.**
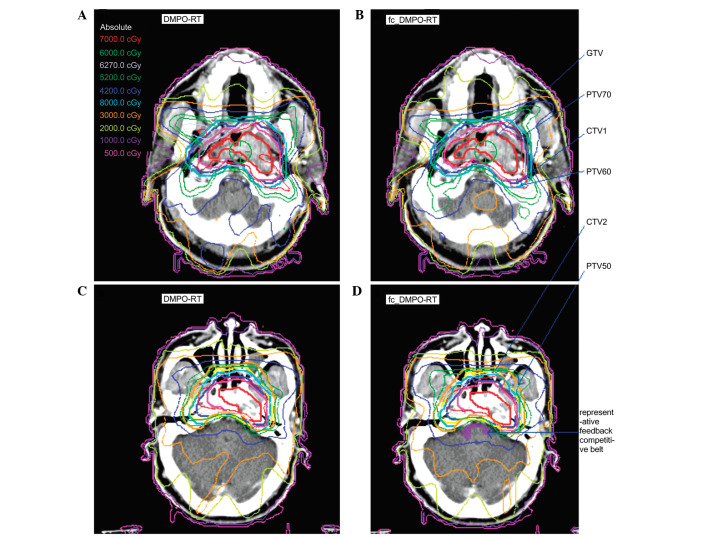
Axial computed tomography images comparing representative isodoses between two planes. (a) DMPO-RT, (b) fc_DMPO-RT (c) DMPO-RT and (d) fc_DMPO-RT. fc_DMPO-RT was better than DMPO-RT, and the representative feedback competitive belt is marked. GTV, gross tumor volume; DMPO-RT, direct machine parameter optimization radiotherapy method; fc_DMPO-RT, feedback constraint DMPO-RT method; PTV, planned target volume; OAR, organs at risk; CTV, clinical target volume.

For the present planning study, the Siemens Primus linear accelerator with a double-focused MLC (1 cm resolution at the isocenter; 27 inner leaf pairs; 6.5 cm for the 2 outer leaf pairs; 6 MV photon energy) was used. Treatment plans utilized synchronous boost technology, comprising 33 fractions with a total dose of 70, 60 and 59.4 Gy for PTV70, mPTV60 and mPTV50, respectively. In these 33 fractions, the sample with a total dose of 59.4 Gy for mPTV50 was virtual and convenient for planning, while 28 fractions with a total dose of 50.4 Gy for mPTV50 was actual. Thus, following irradiation of 28 fractions, treatment plans for the remaining 5 fractions were recalculated by discarding PTV50. The planning study presented here was conducted using the 33-fraction plan. This was considered sufficient for assessment of the quality of the optimization strategy. The dose constraints of the target and OAR in the plan were V_95%_ of PTV≥98% and V_110%_≤10%, (V_x%_, volume percentage of planning target with X% indicating prescription dose received); D_1cm_^3^ of the spinal cord (1 cm^3^ volume maximum dose) <42 Gy; D_1cm_^3^ of the brainstem <52 Gy; D_50%_ of the parotid gland (dose on 50% volume) <26 Gy; D_1%_ (dose on 1% volume) of the optic nerve and optic chiasm <52 Gy; and D_1%_ of the eye lens as low as possible (≤5 Gy). Two plans were developed for each patient using the two methods outlined, resulting in a total of 46 plans for the 23 NPC patients. The 2 types of plan were designed as follows: i) For the DMPO-RT, 7 coplanar fields with angles of 280°, 240°, 210°, 180°, 150°, 120° and 80° at the lower section of the head were chosen in order to locate the mouth and eyes, which are low tolerance organs, at the end of the exposure path. Based on the current situation at the Sir Run Run Shaw Hospital, the parameter in the TPS defining the maximum segment number for optimization was set to 80, the smallest segment area was set to 8 cm^2^ and the minimum MU was set to 8 (segments with less jaggedness, larger area and larger minimum MUs had better therapeutic accuracy). The DMPO-RT plan was obtained following optimization based on the above constraint conditions, and the iteration number was set to 80. The segment and iteration numbers were set as high as possible, in order to promote the program optimization process and thus improve plan quality. ii) For the fc_DMPO-RT, all the conditions for the optimization design method were identical to those for DMPO-RT, except the segment number was set to 40–45, and the iteration number was set to 40. Following the above optimization, the iteration number was reduced to 20 and the dose distribution deviation (hot or cold regions) was delineated. Furthermore, certain key areas known as competitive belts (for example, the joint areas between the brainstem and target, where the brainstem is in proximity to the target) are in dose competition with each other; therefore, they have a specific mutual geometric and topological structure, which must be identified and characterized (Fig. 1). Additional competitive belts exist, including those between the parotid gland and mPTV60 and between the optic chiasm and PTV. These competitive belts were also delineated during treatment planning, defined as constraint conditions and fed back into the original constraint condition. These delineated parameters were given varying weights, based on priority. The following optimization was operated by the iteration set to 20. This kind of optimization by feedback constraints was able to be operated 2–4 times, depending on planning complexity. The iteration number could also be reduced following a decrease in the complexity of the feedback condition.

#### Plan evaluation and comparison

The comparison of dose quality for each plan was based on the dose distribution and dose-volume histogram (DVH). Based on the approximate maximum dose of D_2%_, the approximate minimum dose of D_98%_ and the median dose of D_50%_ outlined in the ICRU report 83, as well as the homogeneity index (HI) and conformity index (CI), the dose distribution on the target was assessed, where HI=D_5%_/D_95%_ (D_5%_ and D_95%_ are the doses covering 5% and 95% of the PTV, respectively). The higher the value of the HI, the less homogeneous the plan. CI was calculated as CI=VT_ref_/VT × VT_ref_/V_ref_ (VT, target volume; VT_ref_, target volume covered by the reference isodose and V_ref_, total volume covered by the reference isodose); the higher the CI value, the better the conformance of the plan. D_1cm_^3^ and D_mean_ were computed to evaluate the spinal cord and brainstem; D_1%_ and D_mean_ were used to evaluate the optic nerve, optic chiasm and eye lens; and D_50%_ was employed to evaluate the parotid gland, where D_1cm3_ and D_1%_ were the doses for 1 cm^3^ and 1% of OAR volume, respectively and D_mean_ was the average dose.

#### Comparison of plan execution efficiency and dose accuracy

The two kinds of plans were compared in terms of segment number, MUs and delivery time. Delivery time was calculated using a tested formula (11) according to the number of gantry angles, the number of segments and the number of MUs for a Siemens machine with static IMRT: Delivery time (sec)=(segment number-1)×9+(total MUs/dose rate)×60+(number of gantry angles-1)×13. The 2-dimensional diode array detector verification system (MapCheck; Sun Nuclear Corp., Melbourne, FL, USA) was used to test the dose accuracy of the 2 types of plan.

#### Statistics

Normally distributed data are presented as the mean ± standard deviation. A paired *t*-test, using SPSS 13.0 software (SPSS Inc., Chicago, IL, USA), was performed to analyze the two plans. P<0.05 was considered to indicate a statistically significant difference.

## Results

### 

#### Comparison of dose distribution to the target

Compared with DMPO-RT, fc_DMPO-RT resulted in a more rational PTV70 dose distribution. Furthermore, the dose and minimum dose (D_98%_) were increased with fc_DMPO-RT, resulting in a significant improvement in local tumor control. Thus, it was hypothesized that feedback constraint may supplement the cold point in PTV70 and improve the conformity of PTV70. With feedback constraint, the distribution of the total dose volume was enhanced by slightly increasing the value of the MUs, which were in the normal fluctuation range of the prescribed PTV70 dose. Areas around the competitive belt, close to the PTVs, had a tighter dose gradient and isodose contour distribution (Fig. 1). The dose of mPTV60 also increased, but this was not as marked a change as that observed in the PTV70, and there was no statistically significant increase in mPTV50. In addition, there were no statistically significant differences in HI and CI (Table II and Fig. 1).

#### Comparison of dose distribution to the OAR

**Figure 2.**
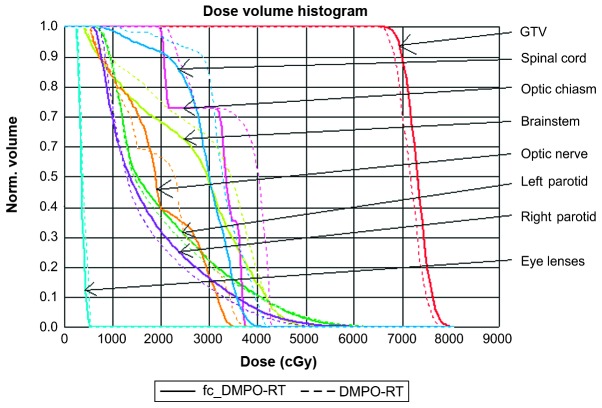
Representative dose-volume histogram from the 2 types of plan, with 33 fractions based on 2 methods. m planned target volume 50 was a virtual prescription of 59.4 Gy for 33 fractions, while the actual irradiated dose was 50.4 Gy for 28 fractions. Left parotid, left parotid gland; right parotid, right parotid gland. DMPO-RT, direct machine parameter optimization radiotherapy method; fc_DMPO-RT, feedback constraint DMPO-RT method; GTV, gross target volume.

fc_DMPO-RT resulted in a significant decrease (P<0.05) in the maximum dose to the spinal cord as compared with that of DMPO-RT. There were no statistically significant differences in doses to the brainstem, optic chiasm, optic nerve, parotid gland or eye lenses between the two methods. The dose distribution to the OAR for both methods met the clinical requirements well (Table III). A representative DVH for the 23 patients is depicted in Fig. 2.

#### Comparison of treatment efficiency and dose verification

There was a significant difference in the MUs, total numbers of segments and delivery time between the two methods (*t*=6.2, 393.4 and 244.3, respectively; P<0.05). Delivery time was reduced by 26.8% with fc_DMPO-RT, compared with that of DMPO-RT. However, the MapCheck verification pass ratio indicated no statistical difference between the 2 plans (Table IV).

## Discussion

In addition to DVH and dose assessment, the efficiency of planning execution should be carefully considered in the evaluation of the complex IMRT plan. Although the use of a greater number of segments improves the plan quality, there is a threshold for the maximum number of segments (12,13,15). When the segment number reaches a certain threshold, the plan is unable to be further improved as a larger number of segments will increase the plan complexity and sacrifice the efficiency of plan execution. In addition, a larger number of segments will result in a longer delivery time, thereby decreasing the accuracy of radiotherapy due to the variable changes in patient location and fractional position. In addition, the excessive radiation leakage may increase the risk of secondary cancer, and the biological effects of the treatment would be poor (4,16).

Advancements in complex IMRT planning have continued to improve. A particular method aimed to reduce the total number of segments and MUs with equal plan quality. Bratengeier *et al* (5,17–19) reported that segments should reflect the geometry and topology of the PTV and OAR in the research of plan optimization by segment reduction. Therefore, segments should fit well with the PTV and OAR structures, and consequently, the plan should be more efficient. Accordingly, they proposed to construct the segments prior to optimization. Three types of segment were created: 0 order segments, comprising conformal PTV including the OAR; 1 order segment, to shape PTV with the OAR blocked; 2 order segment, compensation of block-out-losses in the PTV with multi-directions and narrowed to direct towards PTV areas adjacent to the OAR (5,17–21). The use of the 2 order segment may make the dose distribution gradient between the PTV and OAR steeper and more desirable. Based on these 3 types of segment, it was suggested that this DMPO plan required fewer segments than Pinnacle's DMPO plan, while maintaining equivalent or better plan quality. However, the MUs in their DMPO plan were slightly greater than those in Pinnacle's DMPO plan. The IM plan is not comparable to these DMPOs in terms of quality (18); however, it was also suggested that quantitative definition and introduction of the 2 order segment has a key role in determining plan quality (5,17). The DMPO with the initial optimal point of 2 order segments may have a more rapid and improved convergence (5). In the present study, a large segment with less jaggedness was utilized by limiting the smallest segments and MUs, prior to the introduction of the feedback constraint optimization method. This feedback constraint included intensive modulation for the anticipated dose distribution, as well as adaptive modulation for the anatomy and geometry of the PTV and OAR. For example, the feedback intensive modulation on the space between the GTV and brainstem resulted in a steeper dose gradient. The methods demonstrated in the present study may be similar to the aforementioned 2 order segment proposed by Bratengeier *et al* (5,17–21). The present study highlighted that the timely introduction of feedback based on the anticipated dose distribution and the interactive position between the PTV and OAR may have adaptive and intelligent processing, resulting in an improved IMRT plan. In the fc_DMPO-RT method, the segment created to optimally fit with the geometric shape of PTVs and the anatomical structure of OARs was able to better reflect the relative position of each object (including PTVs and OARs) and the dose gradient of competitive belts. Thus, fewer segments may be sufficient for the construction of good conformity between the fc_DMPO-RT plan with the DMPO-RT plan, while achieving similar conformity of the DMPO-RT plan requires a greater number of assembled segments.

A good IMRT plan should have few segments and MUs. However, there is a trade-off between the quality of the plan and the number of segments. Considering the distribution of the OAR and PTV structure and the degree of complexity, a plan requires varying segment numbers. Based on DAO, Jiang *et al* (15) reported that the number of segments should be ≥60 for complex head and neck cancers, while Ludlum and Xia (11) reported that using the Pinnacle DMPO, treatment of NPC requires >50 segments. In the present study, the treatment plan for NPC required ~40 segments to optimize the plan and meet clinical requirements.

In the DMPO-RT plan, the TPS parameter of the maximum number of segments was set to 80, which was considered sufficient for optimization. The optimizer had no restriction on the maximum number of segments and automatically consumed sufficient segments in every plan to build the perfect plan. Nevertheless, the number of segments in the DMPO-RT plan did not exceed 80. If identical maximum numbers of segments were set in the DMPO-RT plan as in the fc_DMPO-RT plan, the optimized DMPO-RT plan quality would not be comparable with that when the plan was set to 80 maximum segments. The fc_DMPO-RT plan, which incorporated feedback constraint optimization, may obtain improved quality with 40 segments than that of the DMPO-RT plan with 80 segments, thereby meeting the clinical treatment requirements.

In planning studies for head and neck cancer, a primary concern has been regarding how to reduce MUs as much as possible. Table V lists several studies using the DMPO method with DSS radiation technology. The MUs in the present study were similar to those reported in studies by Dobler *et al* (10) and Ludlum and Xia (11). However, the MUs reported in the present study were greater than those reported by Oliver *et al* (22). Therefore, MUs may be relative to the TPS version, cancer site or beam setup; and a more detailed discussion of this topic is outside the scope of this article.

The plan delivery time is not only proportional to the number of segments and MUs of the plan, but is also dependent on the accelerator machine performance, including gantry angling and segment formation times; if the former is larger than the latter, gantry angling will consume more time during treatment and the delivery time will be increased.

There were certain limitations to the present study. Bratengeier *et al* (5,17) reported that the number of segments may be decreased with an increase in beam angles and that plan quality is able to be maintained or even improved. Therefore, for the static IMRT methods of treatment of NPC proposed in this paper, 7 angles may be insufficient, and the application of ≥9 beam angles in IMRT may be evaluated by further study. Furthermore, there is no strict mathematical proof or logical reasoning behind the methods of optimization presented in the current study. In addition, the present method only addressed optimization of the IMRT plan for NPC patients, but may also apply to complex IMRT planning for other carcinomas, including prostate and breast cancer. Finally, rather than developing a novel in-house planning system, the method used in the current study employed Pinnacle, a commercial TPS, to illustrate the methods of feedback constraint and its effectiveness.

Although intensity-modulated arc therapy and volumetric-modulated arc therapy have advantages over IMRT in terms of exposure time, IMRT remains the gold standard strategy for a complex treatment plan, for example that required for the treatment of NPC. It is important to optimize the IMRT plan with fewer segments and MUs while maintaining or even increasing the plan quality. An optimized IMRT plan incorporating these criteria may improve the therapeutic ratio and reduce treatment time, resulting in an improved machine utilization rate with more patients treated per hour. Therefore, we propose that the fc_DMPO-RT method is promising and should be considered for IMRT planning.

## Figures and Tables

**Table I.** Patient characteristics (n=23) and planning target volumes in the present study.

No.	Gender	Age, years	Pathology	Stage	PTV70, cm^3^	mPTV60, cm^3^	mPTV50, cm^3^
1	M	68	Nk carcinoma	T4N1M0	196.2	552.8	244.0
2	M	65	Udf carcinoma	T1N2M0	154.5	637.4	399.6
3	F	57	Nk carcinoma	T2N2M0	123.1	355.0	426.7
4	M	56	Pdf squamous carcinoma	T4N1M0	206.6	83.5	612.0
5	M	58	Nk carcinoma	T4N2M0	192.5	290.7	689.7
6	M	64	Nk carcinoma	T2N0M0	120.1	507.6	467.5
7	M	52	Nk carcinoma	T2N2M0	216.0	342.1	117.3
8	F	61	Udf carcinoma	T2N1M0	91.4	285.7	549.2
9	M	77	Udf carcinoma	T2N2M0	119.5	128.6	538.3
10	M	49	Udf carcinoma	T2N2M0	123.6	583.9	311.1
11	M	67	Udf carcinoma	T2N1M0	248.1	467.2	830.3
12	F	56	Nk carcinoma	T2N2M0	196.5	243.3	411.3
13	M	54	Nk carcinoma	T3N0M0	149.8	492.1	295.3
14	M	64	Nk carcinoma	T3N1M0	141.8	662.4	407.2
15	M	48	Pdf squamous carcinoma	T4N1M0	135.5	438.4	363.4
16	M	59	Nk carcinoma	T4N1M0	280.5	348.9	349.2
17	M	63	Nk carcinoma	T4N1M0	140.7	621.3	346.1
18	M	36	Pdf squamous carcinoma	T1N1M0	57.3	477.6	362.4
19	F	59	Udf carcinoma	T2N0M0	62.6	290.0	263.9
20	M	52	Udf carcinoma	T2N1M0	196.4	679.2	183.9
21	M	36	Pdf squamous carcinoma	T2N3M0	173.1	478.9	886.9
22	M	46	Udf carcinoma	T3N1M0	153.5	484.7	199.6
23	M	41	Udf carcinoma	T3N2M0	382.8	269.2	423.6

M, male; F, female; Udf, undifferentiated; Nk, non-keratinized; Pdf, poorly differentiated; PTVn, planned target volume of prescription n Gy; mPTV50, PTV50 truncated where PTV60 and PTV70 overlap with PTV50; mPTV60, PTV60 truncated where PTV70 overlaps with PTV60.

**Table II.** Dose distribution parameters and comparisons of PTV70, mPTV60 and mPTV50 for the two plans (n=23 per group).

Target	Plan	D_50%_, cGy	D_2%_, cGy	D_98%_, cGy	HI	CI
PTV70	fc_RT	7340.1±107.0^a^	7903.4±168.8^b^	6767.5±197.1^c^	1.1±0.0	0.5±0.1
	RT	7139.0±58.2	7695.5±141.5	6417.3±298.8	1.2±0.0	0.4±0.1
mPTV60	fc_RT	6212.6±91.5^d^	7026.3±150.7	4767.6±531.8	1.3±0.1	0.4±0.1
	RT	6127.2±68.1	6959.7±92.6	4700.8±538.8	1.3±0.1	0.4±0.1
mPTV50	fc_RT	5102.6±209.5	5692.7±88.4	3596.2±1272.3	1.5±0.4	0.5±0.1
	RT	5041.8±172.4	5639.9±121.3	3540.2±1262.7	1.5±0.5	0.5±0.1

fc_RT, feedback constraint direct machine parameter optimization method; RT, direct machine parameter optimization method; D_50%_, median dose; D_2%_, maximum dose; D_98%_, minimum dose; HI, homogeneity index; CI, conformal index; PTVn, planned target volume of prescription n Gy; mPTV50, PTV50 truncated where PTV60 and PTV70 overlap with PTV50; mPTV60, PTV60 truncated where PTV70 overlaps with PTV60. Values are expressed as the mean ± standard deviation.

a t=62.7

b t=20.5

c t=22.0

d t=12.8; P<0.01 vs. RT.

**Table III.** Dose distribution parameters and comparisons of doses to the spinal cord, brainstem, parotid gland, optic chiasm, optic nerve and eye lenses for the 2 plans (n=23 per group).

A, Spinal cord, brainstem and parotid gland						

	Spinal cord		Brainstem		Parotid gland D_50%_, cGy	
			
Plan	D_1cm_^3^, cGy	D_mean_, cGy	D_1cm_^3^, cGy	D_mean_, cGy	Left	Right

fc_RT	3907.7±91.3^a^	3178.8±235.0	4790.4±238.7	3475.2±345.0	2304.8±913.1	2006.5±436.4
RT	3982.6±97.1	3241.7±217.2	4698.8±278.1	3463.5±243.5	2248.4±928.7	1890.7±440.8

B, Optic chiasm, optic nerve and eye lenses						

	Optic chiasm		Optic nerve		Eye lenses	
			
Plan	D_1%_, cGy	D_mean_, cGy	D_1%_, cGy	D_mean_, cGy	D_1%_, cGy	D_mean_, cGy

fc_RT	4993.5±1189.8	4168.0±1357.6	4662.7±1511.6	3061.6±1266.6	489.5±46.7	401.6±50.5
RT	4946.0±1128.1	4112.4±1384.2	4453.8±1421.9	3052.5±1329.5	498.0±59.0	409.5±57.4

fc_RT, feedback constraint direct machine parameter optimization method; RT, direct machine parameter optimization method; D_1%_and D_1cm_^3^, maximum dose; D_mean_, average dose; D_50%_, median dose; NPC, nasopharyngeal carcinoma. Values are expressed as the mean ± standard deviation.

a t=7.3; P<0.05 vs. RT.

**Table IV.** Comparison of MUs, segment numbers, delivery time and pass ratio of the 2 plans (n=23).

Plan	MUs, Mu	Segment numbers	Delivery time, sec	Pass ratio, %
fc_RT	992.4±99.1^a^	39.9±1.7^b^	725.6±36.6^c^	94.8±0.7
RT	1054.3±67.0	67.3±6.4	990.6±72.7	94.8±0.8

fc_RT, feedback constraint direct machine parameter optimization method; RT, direct machine parameter optimization method; MUs, monitor units. Values are expressed as the mean ± standard deviation.

a t=6.2

b t=393.4

c t=244.3; P<0.01 vs. RT.

**Table V.** Comparison of data and MUs resulting from various plans.

Study	MUs	TPS	Beams	Cancer site
Present study	992.4±99.0	Pinnacle, V7.6	7, UES	Nasopharyngeal
Ludlum and Xia, 2008	1050	Pinnacle, V7.6	7, UES	Nasopharyngeal
Dobler *et al*, 2009	944±160	Oncentra masterplan, V1.5	9, ES	Oropharyngeal
Oliver *et al*, 2012	711.6±68.1	Pinnacle, V9.0	7, ES	Larynx; tonsil; base of tongue; oropharynx; hypopharynx

MUs, monitor units; TPS, treatment planning system; UES, unequal space; ES, equal space. Values are expressed as the mean ± standard deviation.
